# A Random Forest Algorithm for Assessing Risk Factors Associated With Chronic Kidney Disease: Observational Study

**DOI:** 10.2196/48378

**Published:** 2024-06-03

**Authors:** Pei Liu, Yijun Liu, Hao Liu, Linping Xiong, Changlin Mei, Lei Yuan

**Affiliations:** 1 Department of Mathematics and Physics Second Military Medical University Shanghai China; 2 Department of Health Management Second Military Medical University Shanghai China; 3 Faculty of Health Service Second Military Medical University Shanghai China; 4 Nephrology Department Shanghai Changzheng Hospital Shanghai China

**Keywords:** chronic kidney disease, random forest model, risk factors, assessment

## Abstract

**Background:**

The prevalence and mortality rate of chronic kidney disease (CKD) are increasing year by year, and it has become a global public health issue. The economic burden caused by CKD is increasing at a rate of 1% per year. CKD is highly prevalent and its treatment cost is high but unfortunately remains unknown. Therefore, early detection and intervention are vital means to mitigate the treatment burden on patients and decrease disease progression.

**Objective:**

In this study, we investigated the advantages of using the random forest (RF) algorithm for assessing risk factors associated with CKD.

**Methods:**

We included 40,686 people with complete screening records who underwent screening between January 1, 2015, and December 22, 2020, in Jing’an District, Shanghai, China. We grouped the participants into those with and those without CKD by staging based on the glomerular filtration rate staging and grouping based on albuminuria. Using a logistic regression model, we determined the relationship between CKD and risk factors. The RF machine learning algorithm was used to score the predictive variables and rank them based on their importance to construct a prediction model.

**Results:**

The logistic regression model revealed that gender, older age, obesity, abnormal index estimated glomerular filtration rate, retirement status, and participation in urban employee medical insurance were significantly associated with the risk of CKD. On RF algorithm–based screening, the top 4 factors influencing CKD were age, albuminuria, working status, and urinary albumin-creatinine ratio. The RF model predicted an area under the receiver operating characteristic curve of 93.15%.

**Conclusions:**

Our findings reveal that the RF algorithm has significant predictive value for assessing risk factors associated with CKD and allows the screening of individuals with risk factors. This has crucial implications for early intervention and prevention of CKD.

## Introduction

Chronic kidney disease (CKD) is characterized by chronic structural and functional impairment of the kidney of >3 months, caused by various factors. CKD is diagnosed based on the presence of pathological injury for more than 3 months, abnormal glomerular filtration rate (GFR), abnormal blood or urine composition, abnormal imaging findings, or an index estimated GFR (eGFR) of <60 mL/minute/1.73 m^2^ [1]. CKD is a major global health concern. Between 1990 and 2015, the annual mortality rate attributed to CKD increased at an average rate of 3.4% per year, and the global prevalence rate of CKD increased to 14.3% [2]. The economic burden due to CKD accounts for 31.4% of the global annual burden of living with disability [3-6]. In China, the prevalence of CKD among patients aged 18 years and older is 10.8%, encompassing approximately 120 million patients, indicating that approximately 1 in 10 Chinese individuals have had CKD [1]. Nevertheless, the awareness rate of CKD is low, and only 12.5% of patients know about their illness. CKD is highly prevalent and its treatment cost is high but unfortunately remains unknown. Therefore, early detection and intervention can mitigate the treatment burden on patients and decrease disease progression.

In recent years, risk factors including hypertension, diabetes, and obesity, which are associated with CKD, have gradually shown a trend toward affecting the younger population [7]. CKD is closely linked with an increased risk of all-cause mortality, cardiovascular disease (CVD), renal failure, and other adverse health outcomes, causing a serious disease burden [8-10]. CKD is a major health concern due to its high prevalence, low awareness rate, high treatment cost, increased risk of combined cardiovascular events, and early mortality. Early intervention, treatment, and controlling the risk factors of CKD can decelerate and decrease disease progression and consequently reduce overall morbidity and mortality. Hence, diagnosis and risk factor assessment for patients with early-stage CKD are of immense significance.

With continuous advancements in artificial intelligence technology, many researchers have attempted to use machine learning models in the medical field. Many studies have reported that machine learning algorithms can improve the decision-making abilities of clinicians in different fields, including clinical prediction. A study published in *The Lancet* [11] developed a feasible and effective machine learning–based risk stratification model for predicting adverse events post hospital discharge in patients with acute coronary syndromes. The random forest (RF) algorithm was first proposed by Leo Breiman and Adele Cutler in the early 21st century [12]. In the last few years, the use of the RF algorithm for disease risk prediction has garnered increasing attention due to its high accuracy. Furthermore, some researchers have used econometric models based on logistic regression (LR) and RF to predict the risk of acute ovarian failure [13]. Additionally, Let et al [14] constructed an RF model to improve the early detection and prediction of the incidence of venous thromboembolism in patients with lung cancer.

Some researchers have explored the application of machine learning algorithms in disease prediction, compared them with traditional statistical regression models, and reported the differences in the performance of various prediction models. While comparing conventional LR models with the RF algorithm, many studies reported that the RF algorithm is more advantageous than the LR model. A previous study investigated the predictability of the RF algorithm, the LR model, and deep neural network models and found that machine learning models, particularly deep neural network models, can improve the long-term prognosis prediction of patients with ischemic stroke [15]. Another study constructed an interpretable RF model to predict severe acute pancreatitis and found that the RF model showed better precision and diagnostic accuracy than the LR and Bedside Index Of Severity In Acute Pancreatitis models [16]. Some researchers used 5 machine learning algorithms separately to predict the malnutrition status of 5-year-old children in Bangladesh and found that the accuracy of the RF algorithm was 68.51%, which was greater than that of other algorithms [17]. Another study reported that the RF algorithm is a better predictive model for older patients with hip fractures and high-risk mortality within 1 year after surgery [18].

A longitudinal study involving 143,043 patients with hypertension was performed to predict long-term CVD risk. The study reported that advanced machine learning algorithms using RF performed better than traditional LR [19]. A longitudinal cohort study compared clinical risk predictions among patients with CVD using 19 prediction techniques. The study also reported that excluding LR and commonly used machine learning algorithms from long-term risk prediction models underestimated the disease risk [20].

Researchers have also investigated the advantages of using RF models in predicting kidney diseases. A previous study reported the performance of 4 prediction tools, namely deep learning, plain Bayesian, RF, and LR, for predicting all-cause mortality in patients with CKD. The study showed that Bayesian networks and LR showed superior prediction abilities [21]. However, another study reported that plain Bayesian, RF, and LR performed adequately well and showed high sensitivity for screening end-stage renal disease in patients with CKD, which is inconsistent with previous reports [22]. Another previous study constructed 3 algorithms, namely RF, plain Bayesian, and LR, to classify glomerular and tubular injury and found that RF showed the best performance in terms of accuracy, sensitivity, and specificity. These findings suggest that RF can facilitate early diagnosis of glomerular and tubular injury to mitigate CKD progression [23]. Therefore, previous studies on the viability of RF models have reported inconsistent conclusions due to differences in research perspectives and subjects.

## Methods

### Data Source

The data for this study were collected from the CKD screening population in Jing’an District from January 1, 2015, to December 22, 2020. Information obtained included demographic and sociological characteristics, height, weight, diastolic and systolic blood pressure, health insurance type, screening date, urinary protein and urinary albumin-creatinine ratio (UACR), blood creatinine, eGFR, and screening results. In total, 103,960 records were initially screened and CKD diagnoses were categorized based on *ICD-10* (*International Statistical Classification of Diseases, Tenth Revision*) criteria. Records with incomplete or duplicate data were excluded, resulting in a final sample size of 40,686 cases for analysis. These data are considered credible and authentic.

### Definition of Grouping

The participants were categorized based on dichotomous variables: 1 for the nonmanagement population (indicating the absence of CKD) and 2 for the management population (indicating the presence of CKD).

### Covariance

We used the 11 factors identified in the univariate analysis as explanatory variables for the LR model. The grouping and assignment of the dependent and independent variables are listed in Table 1.

**Table 1:** Grouping and assignment of dependent and independent variables.

Name	Variable	Value assignment
CKD^a^ screening	Y	1. Nonmanagement population; 2. Management population
Gender	*x*_1_	1. Male; 2. Female
Age	*x*_2_	1. <65 years; 2. 65-75 years; 3. ≥75 years
BMI	*x*_3_	1. Normal: 18.5-24; 2. Underweight: <18.5; 3. Overweight: 24-28; 4. Obesity: ≥28
History of hypertension	*x*_4_	1. No; 2. Yes
Index blood creatinine	*x*_5_	1. Normal; 2. Abnormal
Index eGFR^b^	*x*_6_	1. No; 2. Yes
Index urinary protein	*x*_7_	1. Negative; 2. Positive
Albuminuria	*x*_8_	1. No; 2. Yes
Urine albumin- creatinine ratio	*x*_9_	1. <30; 2. 30-300; 3. ≥300
Working status	*x*_10_	1. Retired staff; 2. Unemployed person; 3. Others^c^
Type of medical insurance	*x*_11_	1. Urban employee medical insurance; 2. Urban resident medical insurance; 3. Others^d^

^a^CKD: chronic kidney disease.

^b^eGFR: estimated glomerular filtration rate.

^c^Others include students, freelancers, and workers.

^d^Others include the poverty relief system, out-of-pocket insurance, new rural cooperative medical system (NRCMS), commercial medical insurance, and free medical service. The same as below.

### Statistical Model

A database was established using Excel (Microsoft Corp) 2010, and SAS (version 9.4; SAS Institute Inc) statistical software was used for data analysis. The chi-square test was performed for 1-way analysis to select variables for inclusion in the model, with the threshold for statistical significance set at *P*<.05. Based on the GFR stage, albuminuria (Alb) grouping, and the distribution of data, the study categorized participants for CKD screening into management (suspected and diagnosed patients) and nonmanagement (healthy individuals) populations. The resulting dichotomous LR model was then used for subsequent analysis.

### The RF Algorithm

RF is a classification algorithm that uses multiple decision trees to train and predict samples. Specifically, the algorithm samples the training data set N times with replacement and selects a random subset of training samples each time. The remaining undrawn samples are subsequently used to evaluate the prediction error of the model.

### Training Validation Split

The data set of 40,686 participants was randomly split into the following 2 subsets using simple random sampling in Python 3.6: one for validation sample set A including 13,549 cases (or 33.3% of the total data set), and the other for then training sample set B including 27,139 cases (or 66.7% of the total data set). The first subset A constituted the external validation sample set with 3000 cases (accounting for 7.4% of the total data set). The RF algorithm was subsequently applied to the training sample set to evaluate the importance of each variable and construct a CKD risk factor model. This model was used to predict the test sample set, with a minimum prediction accuracy threshold of 70%.

### Parameters

The mean number of feature selections was used for each random tree (mtry) in the model.

For a set with predictors, a typical number is the rounded square root of mtry [12]. Only 11 features were used in this study. We did not use the square root method to calculate mtry. However, we randomly selected a certain number of features each time and fixed ntree to adjust mtry to determine the values that minimized generalization errors as the optimal value of mtry.

The mean number of random trees was used in the RF algorithm (ntree) in the model. (1) Using bootstrap resampling, 20% of the B set was randomly split and was used as an internal validation set and 80% was used as the training set. (2) Assuming that the number of the decision tree was ntree, for each node, mtry features were randomly selected. These mtry features were used to divide the sample set, and the index Gini was used to determine the best partitioning method. (3) For determining the mean error of the test set, steps (1), (2), and (3) were repeated. With each iteration of step (2), the ntree was increased by 1. ntree gradually increased from 1 to 200. We obtained the set for average generalization error, and observed the variation in the average generalization error with ntree. When the optimal model was achieved, we obtained the number of ntrees.

### Variable Importance

After establishing the RF model, it was used for prediction. Given the abundance of trees in the forest, determining which variables have the most significant impact on predictions can be challenging. Fortunately, an important method was used to assess the significance of variables in the model. Specifically, for each variable, in each decision tree of an RF, the decrease in the splitting criterion function (residual squared or Gini index) caused by that variable was measured. The decrease in magnitude for each decision tree was then averaged to determine the importance of the variable. The importance of each feature variable was ranked and plotted in order, resulting in a variable importance plot.

### Ethical Considerations

The Institutional Review Committee Board at Shanghai Changzheng Hospital affiliated with the Naval Medical University approved this study with written consent (No.2016SL020). This observational study analyzed existing data sources, which did not contain any patient-identifiable information. This study did not involve the collection, use, or transmission of individually identifiable data.

## Results

### LR Model With 2 Classifications

#### Results of Single Factor Analysis

An LR model with 2 classifications (CKD and non-CKD) was used for analysis. As shown in Table 2, the results of the univariate analysis indicate a statistically significant distribution of differences in CKD status in the investigated population across 11 variables: gender, age, BMI, history of hypertension, index blood creatinine, index eGFR, index urinary protein, Alb, UACR, working status, and type of health insurance (*P*<.05).

**Table 2:** Distribution and comparison of baseline characteristics among patients diagnosed with CKD^a^.

Variable name			Total participants, n		Management population, n (%)		Chi-square (*df*)			*P* value	
**Gender**								47.43 (1)			<.001
	Male	17,205		16,052 (93.30)							
	Female	23,481		21,473 (91.45)							
**Age (years)**								7811.50 (2)			<.001
	<65	9638		6864 (71.22)							
	65-75	20,156		19,783 (98.15)							
	≥75	10,892		10,878 (99.87)							
**BMI (kg/m^2^)**								220.31 (3)			<.001
	Normal (18.5-24)	19,444		17,545 (90.23)							
	Underweight (<18.5)	1021		936 (91.67)							
	Overweight (24-28)	15,387		14,457 (93.96)							
	Obesity (≥28)	4834		4587 (94.89)							
**History of hypertension**								8.62 (1)			.003
	No	37,513		34,556 (92.12)							
	Yes	3173		2969 (93.57)							
**Index blood creatinine**								62.35 (1)			<.001
	Normal	39,959		36,798 (92.09)							
	Abnormal	727		727 (100)							
**Index eGFR^b^**								1164.79 (1)			<.001
	Normal	16,817		14,603 (86.83)							
	Abnormal	23,869		22,922 (96.03)							
**Urine protein indicators**								387.10 (1)			<.001
	Negative	36,557		33,396 (91.35)							
	Positive	4129		4129 (100)							
**Albuminuria**								519.68 (1)			<.001
	No	35,329		32,168 (91.05)							
	Yes	5357		5357 (100)							
**Urinary albumin-creatinine ratio**								580.49 (2)			<.001
	<30	34,793		31,632 (90.91)							
	30-300	5207		5207 (100)							
	≥300	686		686 (100)							
**Working status**								1471.67 (2)			<.001
	Retired staff	37,406		35,062 (93.73)							
	Unemployed person	204		142 (69.61)							
	Others	3076		2321 (75.46)							
**Type of medical insurance**								111.97 (2)			<.001
	Urban worker	22,909		21,405 (93.43)							
	Urban resident	16,626		15,055 (90.55)							
	Others	1151		1065 (92.53)							

^a^CKD: chronic kidney disease.

^b^eGFR: estimated glomerular filtration rate.

#### Multivariate Analysis

On univariate analysis, variables with statistically significant differences were subjected to multivariate analysis as explanatory variables in binary LR to establish a regression model. The variables were screened using the input method with a significance level of α=.05. The results of the multivariate analysis are presented in Table 3. The risk of CKD was lower in women than in men (odds ratio [OR] 0.909, 95% CI 0.829-0.997). Furthermore, the risk of CKD gradually increased with an increase in age, with people aged 75 years and older (OR 256.759, 95% CI 151.115-436.259) and those aged 65-75 years (OR 20.471, 95% CI 18.209-23.013) being at higher risk than those younger than 65 years. Moreover, individuals with a BMI above the normal range were at a higher risk of CKD. People with a BMI of ≥28 (OR 2.024, 95% CI 1.426-1.733) and those with a BMI of 24-28 (OR 1.572, 95% CI 1.426-1.733) were at a higher risk of CKD than those with a normal BMI. Similarly, people with an abnormal eGFR index were at a higher risk of CKD (OR 1.397, 95% CI 1.271-1.537) than those with a normal eGFR. Compared with other participants, retirees (OR 2.432, 95% CI 2.162-2.736) and people with medical insurance for urban employees (OR 1.769, 95% CI 1.319-2.372) were at higher risk of CKD.

Table 4 shows that in the test sample, a high proportion of records (98.9%) was accurately predicted. Specifically, the prediction model correctly identified all management population records, whereas only 6.4% of nonmanagement population records were accurately predicted.

Although dichotomous LR offers notable advantages including fast training, easy understanding, and high interpretability, its limitations should be acknowledged. First, its effectiveness may be hampered when managing imbalanced data sets, as observed in this study where indicators including urine routine proteins (PROs) exhibited excessive ORs because of the higher proportion of abnormal values within the management population. Second, similar to the accuracy rates of linear models, the accuracy rates of LR models may not be optimal because the latter can experience difficulty in fitting the true data distribution. Herein, imbalanced data sets in the regression model led to statistically insignificant urine test results. Thus, to overcome these limitations, we considered using a machine learning approach.

**Table 3:** Logistic regression analysis of factors affecting chronic kidney disease in people with different characteristics.

Variable name			β		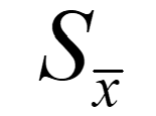				Wald chi-square (*df*)		*P* value				Odds ratio (95% CI)
Female gender (reference: male)			–0.095		0.047				4.103 (1)		.04				0.909 (0.829-0.997)
**Age (years; reference: ≤65 years)**															
	65-75	3.019		0.060				2555.045 (1)		<.001				20.471 (18.209-23.013)	
	≥75	5.548		0.270				420.803 (1)		<.001				256.759 (151.115-436.259)	
**BMI (kg/m^2^; reference: normal [18.5-24 kg/m^2^])**															
	Underweight (<18.5)	–0.286		0.148				3.737 (1)		.05				0.751 (0.562-1.004)	
	Overweight (24-28)	0.452		0.050				82.521 (1)		<.001				1.572 (1.426-1.733)	
	Obesity (≥28)	0.705		0.081				76.341 (1)		<.001				2.024 (1.728-2.370)	
Having a history of hypertension (reference: no)			0.127		0.089				2.031 (1)		.15				1.135 (0.953-1.352)
Abnormal index blood creatinine (reference: normal index blood creatinine)			16.407		1054.200				0.000 (1)		.99				1.33×10^7^ (0.000-0.000)
Abnormal index eGFR^a^ (reference: normal index eGFR)			0.335		0.048				47.630 (1)		<.001				1.397 (1.271-1.537)
Positive urine protein indicators (reference: negative urine protein indicators)			15.990		436.534		0.001 (1)						.97		8.80×10^6^ (0.000-0.000)
Having albuminuria (not having albuminuria)			17.360		403.317		0.002 (1)						.97		3.46×10^7^ (0.000-0.000)
**Urine albumin-creatinine ratio (reference: <30)**															
	30-300	17.435		440.654		0.002 (1)						.97		3.73×10^7^ (0.000-0.000)	
	≥300	15.824		1063.960		<0.001 (1)						.99		7.45×10^6^ (0.000)	
**Working status (reference: other)**															
	Retired staff	0.889		0.060		218.852 (1)						<.001		2.432 (2.162-2.736)	
	Unemployed person	–0.032		0.203		0.026 (1)						.87		0.968 (0.651-1.441)	
**Type of medical insurance (reference: other)**															
	Urban employee medical insurance	0.570		0.150		14.504 (1)						<.001		1.769 (1.319-2.372)	
	Urban resident medical insurance	–0.159		0.151		1.116 (1)						.29		0.853 (0.634-1.146)	

^a^eGFR: estimated glomerular filtration rate.

**Table 4:** Classification of model predictions.

Real test		Prediction of chronic kidney disease status				Percentage of accurate predictions, %
		Nonmanagement population, n		Management population, n		
**Chronic kidney disease**						
	Non-management target population	3	44		6.4	
	Management target population	0	3818		100.00	
Total percentage						98.90

### Machine Learning: RF Algorithm

#### Modeling

**Figure 1:**
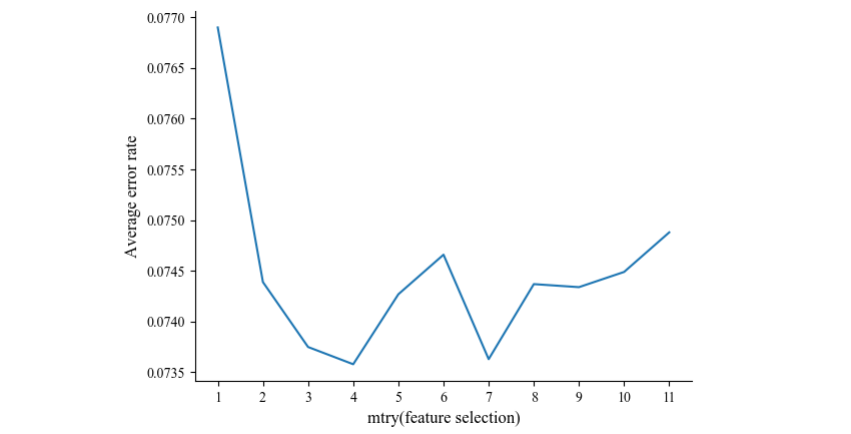
The effect of mtry on the error rate of random forest algorithm.

**Figure 2:**
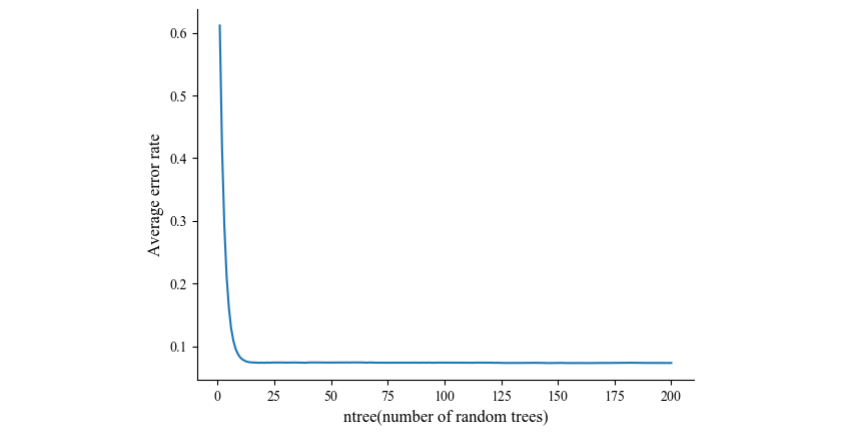
The effect of ntree on the error rate of the random forest (RF) algorithm.

The data set was split into 66.7% of samples, which corresponded to 27,139 records, randomly selected without replacement. The control method was applied by fixing the ntree (number of means of random trees in the RF algorithm) constant and debugging the mtry (mean number of feature selections used for each random tree) parameter. In each iteration, a certain number of features were randomly selected, and the average generalization error value was computed for 11 trials. The change in the error rate of the model, with respect to mtry, is depicted in Figure 1. The error rate decreased significantly when the number of features changed from 1 to 2, followed by an increase close to the minimum value, which was achieved when mtry=4. Next, the mtry value was set to 4, and the ntree value was adjusted accordingly. In total, 200 random trials were conducted to gauge the average generalization error of the test set (Figure 2). The generalization error rate decreased rapidly from 1 to 10, decreased slowly from 10 to 25, and thereafter flattened and stabilized. Thus, the optimal model was identified when the ntree value was 166.

#### Analysis of the Results of the RF Algorithm

The RF algorithm was trained on a test data set comprising 27,139 records, with ntree=166 and mtry=4. Using these parameters, the algorithm was applied to classify the test set data, and the importance ranking of each feature was determined (Multimedia Appendix 1). The 4 most important features identified were age, Alb, working status, and UACR. These features were further selected for the prediction study, which yield a final classification accuracy rate of 92.67%.

**Figure 3:**
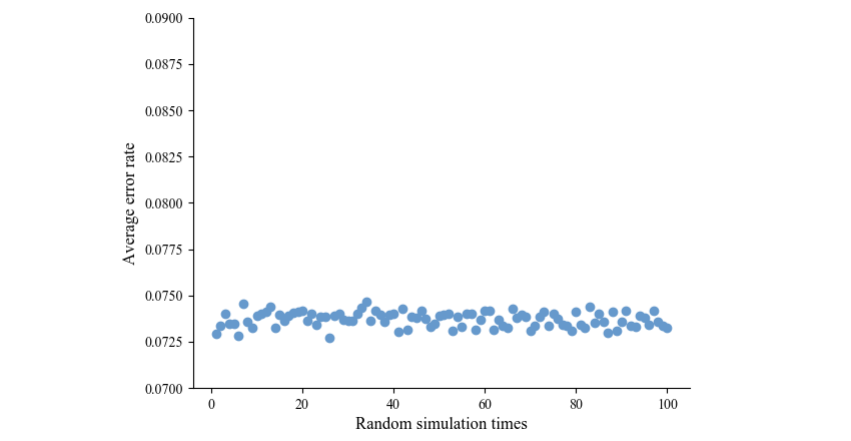
The generalization error rate of the random forest algorithm was estimated by conducting 100 randomized trials.

Next, 100 random trials were conducted to ensure the reliability of our results. The generalization error plot is presented in Figure 3. The error was concentrated around 0.0735, with a small fluctuation and an average error of 7.371%. Our results indicate a good generalization ability of the model, suggesting its reliability in classification tasks.

### Comparison of the Sensitivity and Specificity of RF Models

**Figure 4:**
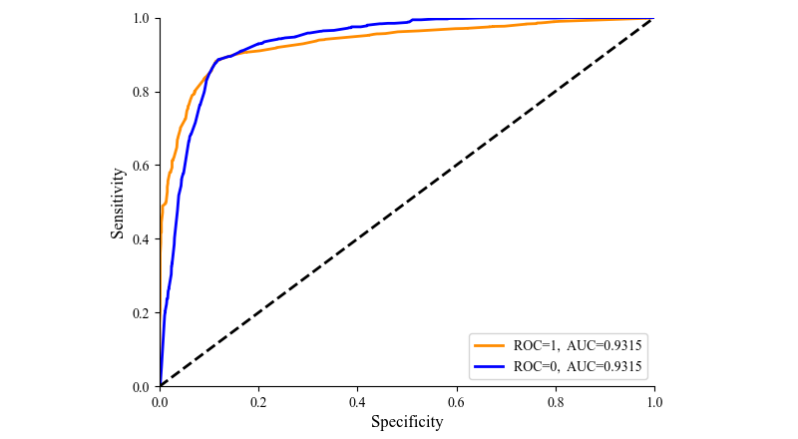
Receiver operating characteristic (ROC) curve of chronic kidney disease prediction by the random forest algorithm. AUC: area under the receiver operating characteristic curve.

The area under the receiver operating characteristic curve (AUC) of the RF model based on the training and test sets was 93.15% (Figure 4). The RF algorithm outputs voting results (0s and 1s), whereas the receiver operating characteristic curve requires voting probability data. Converting probabilities to voting results can lead to error because of extreme probabilities, such as 0.01515526 and 0.98484474. Therefore, we calculated the AUC to assess model performance and the classification prediction rate to indicate the accuracy of the model. Herein, the RF algorithm achieved an accuracy rate of 92.67%, with some degree of error. These results suggest that the model exhibited good predictive power and accurately classified new data samples.

### Confusion Matrix

Four possible predicted results were as follows: true positives, false positives, true negatives, and false negatives. Table 5 shows the confusion matrix of the RF model. The precision, recall, and *F*_1_-score were 0.951, 0.984, and 0.967, respectively.

**Table 5:** Confusion matrix of the random forest algorithm model.

	Predicted values (=1)	Predicted values (=0)
Actual values (=1)	True positive: 12,505	False negative: 209
Actual values (=0)	False positive: 640	True negative: 195

## Discussion

### Principal Findings

A risk assessment model for CKD was developed in this study using dichotomous LR and RF models. Our results indicate that gender, older age, BMI beyond the normal range, abnormal index eGFR, retirement status, and urban employee medical insurance were significantly associated with a higher risk of CKD. By leveraging the RF model, the most important factors for CKD development were older age, abnormal urinary test results (eg, Alb, UACR, and index PRO indicators), and high BMI.

In China, the number of studies on the assessment of risk factors for CKD and the investigation of methods for risk prediction is increasing and LR analysis is commonly being performed. Feng et al [24] used an adjusted LR model to investigate CKD prevalence and related risk factors in 38 megacities across China. Liu et al [25] and Yang et al [26] performed cross-sectional studies to analyze risk factors for diabetic nephropathy in Shanghai, whereas a community-based, 7-year-long cohort study from Tianjin used LR to examine the association between the high triglyceride waist phenotype and risk of CKD development [27]. Yan et al [28] performed LR analysis to assess the correlation between residual cholesterol levels and CKD, and identify other significant risk factors affecting middle-aged and older individuals residing within a city. Gradual advancements in machine learning models have prompted further scrutiny of the divergent performance and inherent limitations of the conventional LR approach. To distinguish this study from previous studies that followed the LR approach for exploratory purposes, we used the RF algorithm to rank risk factors that were subjected to single-factor analysis according to their relevance and consequently evaluated comparative predictive precision by performing LR analysis using training samples. Our results reveal that both the RF and LR models achieved an overall accuracy rate exceeding 90% in the prediction test set. Conversely, the dichotomous LR model exhibited a marginally superior predictive performance than the RF model. Nevertheless, one should pay attention to the tendency of LR to result in excessive ORs when imbalanced data are used. Although LR exhibits excellent predictive abilities and desirable attributes such as high accuracy and stability, and ease of operation with a minimal possibility of overlearning during classification prediction, RF has the ability to assess the importance of variables when classifying data into suitable categories while compensating for errors in imbalanced sets of categorical data.

Our results indicate that age was the primary significant factor in the RF model, and LR analysis confirmed that higher age was significantly associated with CKD. Compared to participants aged ≤64 years, those aged 65-75 years and older were at a significantly higher risk of CKD, which is in line with previous results [29,30]. The risk of CKD increases with age; thus, early screening and risk prediction for CKD are crucial for middle-aged and older people.

A cross-sectional study published in *The Lancet* [31], using a nationally representative sample of Chinese adults also identified independent factors associated with kidney damage, which included age and gender. Age and gender are independent CKD risk factors [32]. Many studies worldwide have shown that women are at a higher risk of CKD [33,34], and similar observations have been reported in China [24,30]. This correlation may be attributed to differences in the prevalence of primary diseases and the availability of medical resources across genders [35]. However, our results show that females in the survey population were at a lower risk of CKD than were males, which is inconsistent with the majority of previous results. Our data include information regarding the registered population in a district of Shanghai. The exclusion of samples with incomplete information and regional differences, as well as the presence of unregistered patients, may have led to bias, ultimately yielding inconsistent results.

Next, this study shows that people with a higher-than-normal BMI were at a higher risk of CKD, similar to a time-series study that investigated risk factors regarding CKD burden in China from 1991 to 2011 and identified the correlation between high BMI and CKD [36]. Obesity is an important risk factor for CKD worldwide [24,25,37-39]. Potential obesity-associated factors that may lead to or aggravate CKD include hemodynamic disorder and renal tissue hypoxia [40,41]. However, weight loss through diet and regular exercise can reverse kidney damage; hence, maintaining a healthy lifestyle and controlling body weight could prevent or decelerate CKD progression to a certain extent [42]. Additionally, this study shows that CKD risk was higher in people who had urban employee medical insurance. These people were employed and had relatively better economic conditions; however, health risk factors such as work stress and unhealthy lifestyles probably contribute to an increased CKD risk [43].

Moreover, people with abnormal urine test results (Alb, UACR, and PRO indicators) were at a higher CKD risk, which is consistent with previous results reported worldwide [36,44,45]. Similarly, a Chinese study using 4 machine learning models, comprising 19,270 adult samples, showed that UACR, Alb, age, and gender were important CKD risk factors [44]. Urine tests can serve as an early warning system for CKD detection. Similarly, our risk prediction model could guide decision-making regarding early CKD screening.

### Limitations

Herein, we effectively assessed the risk of CKD by combining internal data for model construction and testing. However, this study has some limitations. First, the generalization ability of the model remains unknown because the study did not include external data for external validation. Second, owing to the bias in data collection, our results were inconsistent with those of the previous studies. Finally, more prospective studies are required to verify the predictive power and practical utility of our model. Thus, health care professionals should routinely evaluate the level of agreement within and between models before reaching any clinical decision on the basis of the present limitations and previous findings [46].

### Conclusions

In conclusion, the RF model has significant predictive value for assessing risk factors associated with CKD and is capable of correcting errors in imbalanced categorical data sets. It can be used to screen individuals with risk factors, which is of great significance for early intervention and prevention of CKD.

For the prevention and treatment of CKD, early intervention can involve a low-protein diet, regular physical examination, actively promoting urine examination, and screening of high-risk groups to achieve early detection, early treatment, early diagnosis, and early intervention of CKD, and to reduce the social and personal losses caused by diseases and improve people’s quality of life.

## Appendix

Multimedia Appendix 1 Importance ranking of each indicator in the random forest algorithm.

## Abbreviations

Alb: albuminuria
CKD: chronic kidney disease
CVD: cardiovascular disease
eGFR: estimated glomerular filtration rate
GFR: glomerular filtration rate
ICD-10: International Statistical Classification of Diseases, Tenth Revision
LR: logistic regression
OR: odds ratio
PRO: urine routine protein
RF: random forest
UACR: urinary albumin-creatinine ratio

## Data Availability

The data sets used or analyzed in this study are available from the first author upon reasonable request.
