# Supplementary figures and images for: A Random Forest Algorithm for Assessing Risk Factors Associated With Chronic Kidney Disease: Observational Study
Source: Asian Pac Isl Nurs J. 2024 Jun 3;8:e48378. doi: 10.2196/48378 (PMC11184270; doi:10.2196/48378)

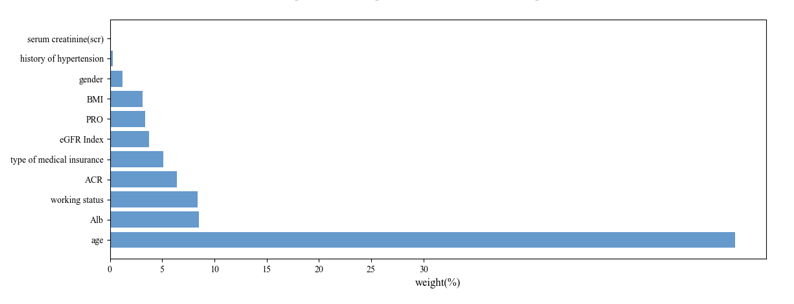

Supplement: Multimedia Appendix 1 [file apinj_v8i1e48378_app1.png]
